# Repetitive transcranial magnetic stimulation as adjunctive treatment in Parkinson’s disease: are we missing opportunities? An umbrella and scoping review

**DOI:** 10.1093/braincomms/fcag346

**Published:** 2026-09-09

**Authors:** Marcelo D Mendonça, Gonçalo Cotovio, Raquel Barbosa, Jean-Pascal Lefaucheur, Joaquim J Ferreira, Albino J Oliveira-Maia

**Affiliations:** Champalimaud Clinical Centre, Digital Neurotherapeutics Centre and Neuroscience of Disease Programme, Champalimaud Foundation, Lisboa 1400-038, Portugal; NOVA Medical School, NMS, Universidade NOVA de Lisboa, Lisboa 1169-056, Portugal; Champalimaud Clinical Centre, Digital Neurotherapeutics Centre and Neuroscience of Disease Programme, Champalimaud Foundation, Lisboa 1400-038, Portugal; NOVA Medical School, NMS, Universidade NOVA de Lisboa, Lisboa 1169-056, Portugal; Department of Clinical Pharmacology and Neurosciences, Parkinson Expert Center, Centre d'Investigation Clinique CIC1436, NeuroToul COEN Center, Toulouse 31300, France; NS-PARK/FCRIN Network, University Hospital of Toulouse, Toulouse 31300, France; UR4391, ENT Team, Faculty of Health, Paris-Est Creteil University, Créteil 94000, France; Department of Clinical Neurophysiology, Henri Mondor University Hospital, AP-HP, Créteil 94000, France; Laboratory of Clinical Pharmacology and Therapeutics, Faculdade de Medicina, Universidade de Lisboa, Lisbon 1649-028, Portugal; CNS - Campus Neurológico, Torres Vedras 2560-280, Portugal; Department of Neurosciences and Mental Health, Neurology, Unidade Local de Saúde de Santa Maria, Lisbon 1649-035, Portugal; Champalimaud Clinical Centre, Digital Neurotherapeutics Centre and Neuroscience of Disease Programme, Champalimaud Foundation, Lisboa 1400-038, Portugal; NOVA Medical School, NMS, Universidade NOVA de Lisboa, Lisboa 1169-056, Portugal

**Keywords:** Parkinson’s disease, rTMS, motor, rehabilitation

## Abstract

Despite the availability of effective treatments, many symptoms of Parkinson’s disease remain insufficiently addressed. Repetitive transcranial magnetic stimulation (rTMS), a non-invasive neuromodulation technique, may help fill these therapeutic gaps, but its clinical role remains unclear. We conducted an umbrella review of systematic reviews and a scoping review of original studies on rTMS for Parkinson’s disease, focusing on motor and non-motor symptoms. Review-level evidence and guideline statements support probable benefit of high-frequency rTMS targeting bilateral motor areas (particularly primary motor cortex) for improving motor symptoms. Low-frequency stimulation of premotor and cerebellar circuits has also shown signals for reducing levodopa-induced dyskinesias, although evidence is less definitive. For non-motor symptoms, high-frequency rTMS of the left dorsolateral prefrontal cortex (DLPFC) appears clearly beneficial for depression, with positive signals for cognitive symptoms, while right-DLPFC stimulation has shown preliminary signals on sleep disturbances. Across most domains, conclusions are limited by small sample sizes, short follow-up periods, heterogeneous stimulation protocols, inconsistent outcome measures and variable methodological quality of both trials and reviews. Evidence therefore remains insufficient to support broad clinical implementation across many symptom domains. Future research should adopt a circuit-based, symptom-specific approach, aligning stimulation targets with the neural substrates of each symptom. This requires clear inclusion criteria, well-characterized patient subgroups and standardized outcome measures across studies. The current literature suggests that rTMS may have therapeutic potential as part of multidisciplinary Parkinson’s disease care, but adequately powered and rigorous trials are needed to determine its clinical utility and define where it may meaningfully address persistent therapeutic gaps.

## Teaser

Mendonça *et al*. report that repetitive transcranial magnetic stimulation shows promising therapeutic signals in Parkinson’s disease, strongest for motor symptoms and depression, but evidence remains limited by small trials, heterogeneous protocols and short follow-up. They conclude that circuit-informed, pragmatic studies are needed to guide clinical integration.

## Graphical Abstract

**Graphical Abstract:**
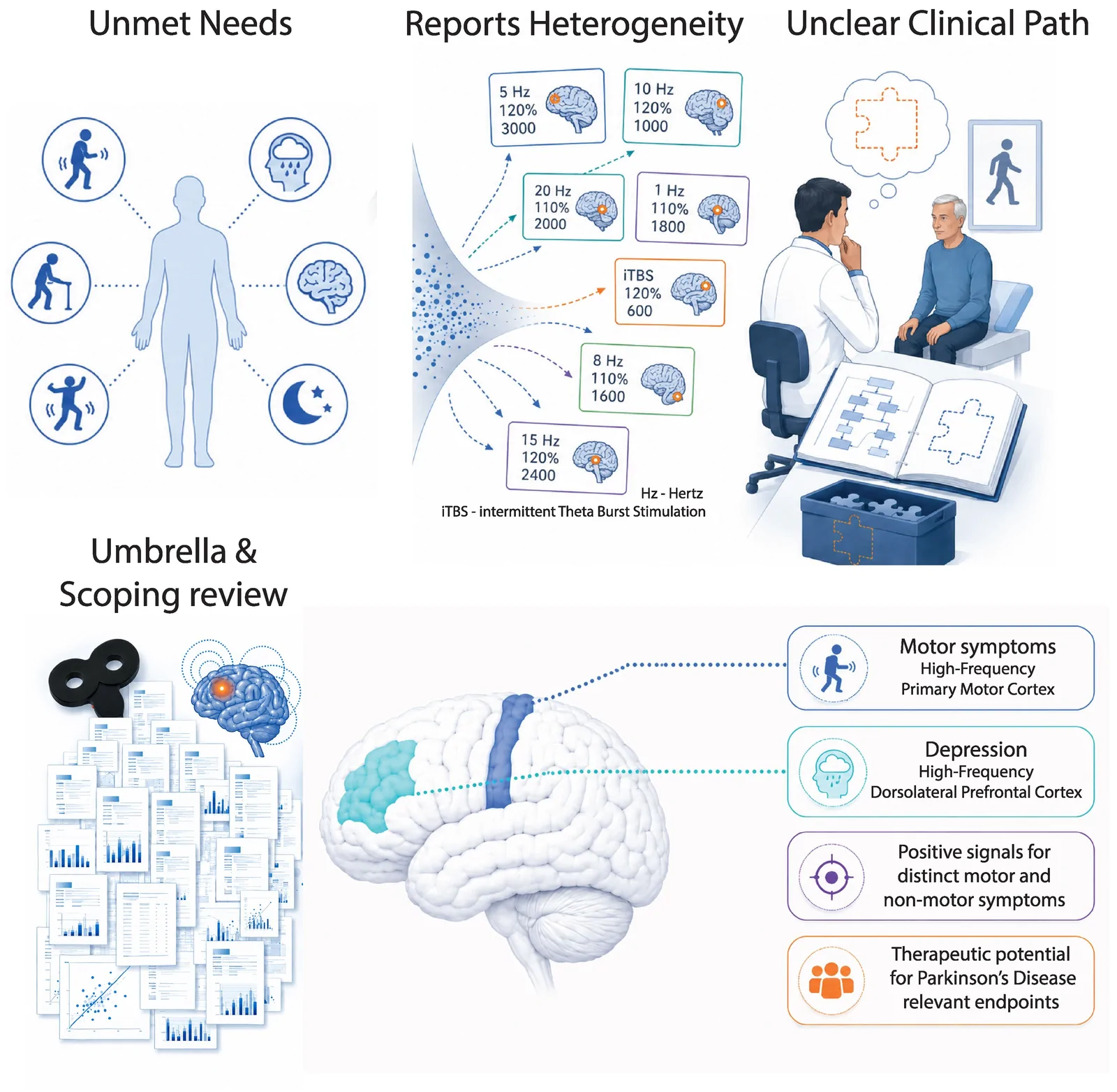

## Background

Parkinson’s disease is not only the second most common but also and fastest growing neurodegenerative disorder worldwide. Estimates suggest that by 2030, 12 million people will be affected globally, rising to 25 million by 2050.^1^ In the absence of disease-modifying therapies, optimizing symptomatic treatments remains crucial and could have a significant impact on disease progression. Yet, despite the transformative effect of levodopa, infusion therapies and deep brain stimulation (DBS),^2^ their benefit is uneven: while some symptoms respond dramatically, others (such as gait, balance, autonomic function, mood, or cognition) show only modest or inconsistent improvement. Movement disorder specialists and people living with Parkinson’s disease continue to confront large areas of unmet need. This makes the exploration of novel strategies, such as neuromodulation targeting symptom-specific brain networks, not just timely but necessary.

During the past decade, the tools available to study and manipulate brain circuits have increased. Evidence has accumulated for several psychiatric disorders that causal brain circuits identified in neuroimaging studies may be the targets for novel strategies of neuromodulation.^3,4^ In Parkinson’s disease, however, the challenge is different: it is a non-remitting, progressive, multisystem disease, where distinct symptoms—even highly correlated ones like rigidity and bradykinesia—map onto partially segregated circuits^5^ and therapeutic needs are distinct at different disease stages. Recent evidence indicates that Parkinson’s disease symptoms can be dissociated at a cortical-circuit level,^6,7^ reinforcing the rationale for tailored neuromodulation strategies.

Repetitive transcranial magnetic stimulation (rTMS) is uniquely positioned in this landscape. It is non-invasive, safe and clinically deployable, with the potential to modulate cortical nodes of basal ganglia–thalamocortical, fronto-limbic and cerebellar loops. rTMS is a non-invasive neuromodulation technique that delivers repeated magnetic pulses to specific cortical regions to modulate neural activity in given brain networks (further details on the technique and sources of variability can be found in Fig. 1). While the 2018 International Parkinson and Movement Disorder Society Evidence-Based update on Treatments for Motor Symptoms^2^ identifies rTMS to only have an investigational role as adjunct therapy, the 2020 evidence-based guidelines on the therapeutic use of rTMS suggest that high-frequency rTMS of bilateral motor regions is probably efficacious on reducing motor symptoms in Parkinson’s disease patients as compared to a sham stimulation.^8^ This apparent gap across clinical recommendations is symptomatic of a broader problem: rTMS is often appraised as a research tool, not as a therapeutic option to be integrated into care pathways.

**Figure 1:**
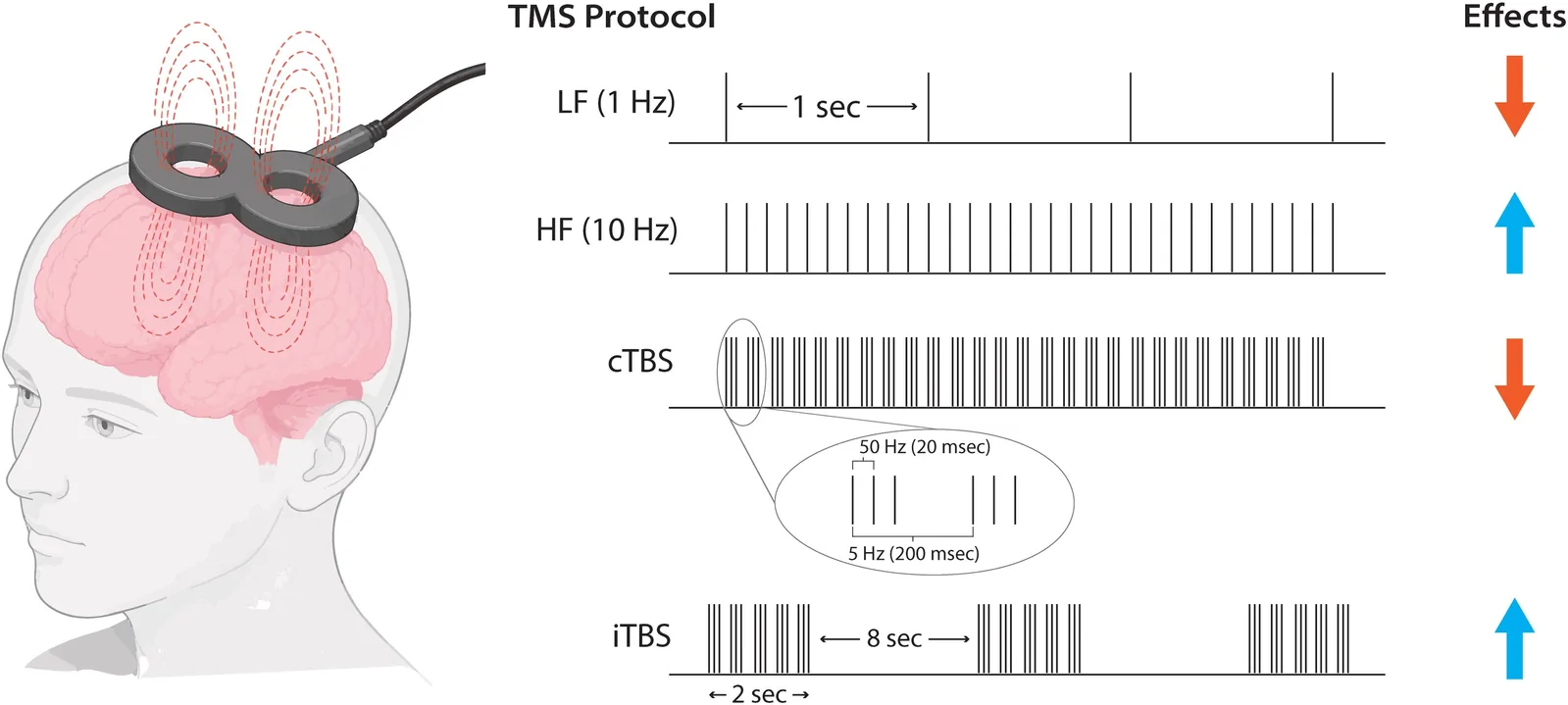
**Repetitive transcranial magnetic stimulation (rTMS) protocols and their effects:** multiple therapeutic protocols have been developed for rTMS, each with potentially distinct effects on neural excitability, as assessed on motor cortex excitability in healthy subjects. Among the most commonly used protocols are low-frequency (LF), high-frequency (HF), continuous theta burst stimulation (cTBS) and intermittent theta burst stimulation (iTBS). Theta burst stimulation (TBS) protocols consist of bursts of three pulses at 50 Hz, repeated at 5 Hz. This can be applied continuously (cTBS), or intermittent application (iTBS). Variability in protocol selection, number of pulses, target regions, number of sessions and stimulation schedules must be considered when interpreting rTMS research and its clinical applications. Although HF rTMS and iTBS are traditionally viewed as excitatory protocols, and LF rTMS and cTBS as inhibitory, this dichotomy is largely based on studies of motor cortex excitability in healthy individuals, where considerable interindividual variability exists. Caution is therefore warranted when generalizing these effects.

There are still multiple unmet needs in the treatment of Parkinson’s disease.^9^ We argue that it is time to reframe rTMS within the perspective of clinical practice. Although numerous systematic reviews and meta-analyses have already catalogued the evidence, what is missing is a synthesis that translates this knowledge into the language of patient care. Beyond summarizing studies, this review therefore provides a narrative framework for how movement disorders specialists might conceptualize rTMS in the clinic: as an adjuvant to therapy and rehabilitation (Fig. 2). We highlight where the evidence is strongest, where methodological gaps remain and how circuit-based thinking can inform the design of future, more practical trials. In doing so, we aim to bridge the persistent divide between research findings and bedside application, and to define how rTMS could contribute to the next generation of Parkinson’s disease therapeutics.

**Figure 2:**
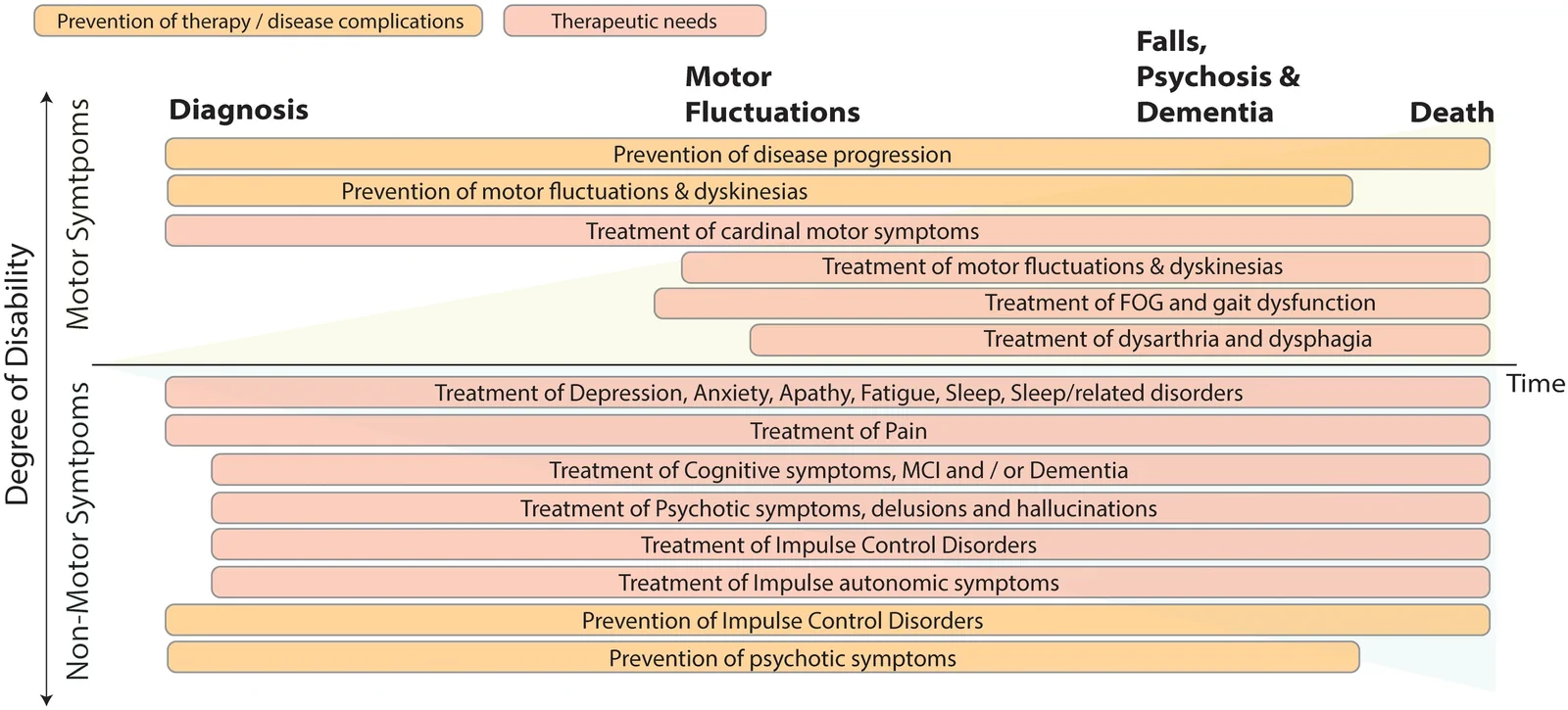
**Conceptual framework for addressing therapeutic needs in Parkinson’s disease.** Parkinson’s disease presents a broad spectrum of motor and non-motor symptoms, with therapeutic needs evolving throughout disease progression. This framework illustrates key areas of intervention, distinguishing between strategies aimed at preventing therapy- or disease-related complications and addressing specific therapeutic needs. Motor symptoms, including fluctuations, freezing of gait (FOG), dyskinesias and dysphagia, become more complex over time, while non-motor symptoms such as cognitive decline, depression, anxiety, psychosis, pain and impulse control disorders also require targeted management. Understanding these evolving needs is essential for optimizing treatment strategies. Given the potential of repetitive transcranial magnetic stimulation (rTMS) to modulate neural circuits involved in both motor and non-motor dysfunction, this framework provides a basis for evaluating current evidence and identifying knowledge gaps to guide future research and clinical applications in Parkinson’s disease. MCI: Mild Cognitive Impairment.

## Methods

We conducted an umbrella review of systematic reviews and meta-analyses on rTMS in Parkinson’s disease, using PubMed searches up to 1 April 2025, with the terms ((systematic review [Publication Type]) OR (meta-analysis [Publication Type])) AND (Parkinson's disease) AND (rTMS). Systematic reviews and meta-analyses were eligible if they evaluated therapeutic rTMS in patients with Parkinson’s disease and reported motor or non-motor clinical outcomes. Broader reviews of non-invasive brain stimulation or non-pharmacological interventions were included when rTMS-specific data for Parkinson’s disease could be extracted. Narrative reviews, protocols, editorials, conference abstracts without full text, non-clinical studies, studies not focused on Parkinson’s disease and reviews in which rTMS-specific effects could not be separated from other interventions were excluded. For symptomatic dimensions with few or no results, we performed a secondary scoping review of original studies using the terms: (clinical trial[Publication Type]) AND (Parkinson's disease) AND (rTMS) AND ((dyskinesia) OR (motor fluctuation) OR (freezing of gait) OR (speech) OR (articul*) OR (dysarth*) OR (dysphag*) OR (deglut*) OR (anxiety) OR (depression) OR (apathy) OR (sleep) OR (RBD) OR (fatigue) OR (pain) OR (cognitive) OR (impulse control disorder) OR (hallucination) OR (delusion) OR (psychotic) OR (behavioural) OR (urinary) OR (autonomic)). Full details are provided in the Results. A final manual search was performed via reference screening to identify any additional relevant studies. Data extraction included review/study characteristics, symptom domain, stimulation target, design, outcomes and main findings. To provide a concise clinical summary, we applied a pragmatic evidence-grading framework informed by the methodological confidence of included reviews (AMSTAR 2) and the risk of bias of randomized trials (RoB 2) summarized graphically using robvis (Risk-of-bias VISualization), a web-based and R-based tool for displaying risk-of-bias judgments. Domain-level and overall RoB 2 judgments were entered into robvis to generate traffic-light plots, allowing visual comparison of bias concerns across studies and domains. Observational and non-randomized experimental studies were considered as supplementary evidence and interpreted cautiously, primarily as hypothesis-generating rather than as a basis for efficacy conclusions. Symptom domains were defined *a priori* using a clinically oriented framework aligned with the conventional organization of Parkinson’s disease treatment literature into motor and non-motor manifestations; differences in the depth of each section reflected the availability of review-level evidence rather than differential prioritization by the authors. +++ indicated consistent evidence of benefit supported by at least one moderate- or high-confidence systematic review/meta-analysis or guidelines, or, when review-level evidence was unavailable, by multiple (≥2) randomized controlled trials at low risk of bias with concordant results and no relevant contradictory results. ++ indicated probable benefit supported by low-confidence review-level evidence or by randomized trials (≥2) showing generally consistent results with some bias concerns. + indicated preliminary support based mainly on only one RCT, open-label studies or non-randomized studies. +/− indicated mixed, inconsistent or insufficient findings. − indicated no clear benefit in the available controlled evidence. A blank entry indicated that no eligible published data were available for that symptom-target pairing.

## Results

The umbrella review search rendered 71 results. A total of 16 reviews were included as they addressed rTMS treatment in Parkinson’s disease and identified specific symptomatic dimensions. Their AMSTAR-2 quality ratings are detailed in Supplementary Table 1. The most frequent methodological limitations were failure to report funding sources of the individual included studies (16/16), absence of a study-level list of excluded full-text articles with reasons (15/16) and limited assessment of the risk of bias on pooled estimates among reviews with meta-analysis (9/12). The scoping review results are addressed in the respective sections.

### Motor symptoms

Data presented on this section are summarized in Table 1.

**Table 1:** Summary of rTMS effects on motor symptoms in Parkinson’s disease

Symptom	M1	SMA	Cerebellum	STG (R)	DLPFC	IFC
Motor symptoms	+++ (HF)	++ (HF) ++ (LF) ++ (cTBS)			+/− (HF) +/− (iTBS)	
Motor fluctuations						
Dyskinesias	+/− (LF 1 Hz)	++ (LF)	++ (cTBS)		+/− (HF)	+ (cTBS)
FOG/Gait	+++ (LF) +++ (HF)	++ (HF)			− (LF)	
Dysarthria	+ (HF)			+ (LF)	+/− (HF)	
Dysphagia	+ (HF)					
Prevention of motor complications						

This table summarizes the current evidence regarding the effects of repetitive transcranial magnetic stimulation (rTMS) on motor symptoms in Parkinson’s disease, highlighting targeted brain regions, stimulation frequencies and evidence of effect on the domain. Protocol types: HF: High-Frequency rTMS (≥5 Hz, commonly 10–20 Hz) LF: Low-Frequency rTMS (typically 1 Hz) cTBS: Continuous Theta Burst Stimulation, iTBS: Intermittent Theta Burst Stimulation Targets: M1: Primary Motor Cortex SMA: Supplementary Motor Area, STG (R): Right Superior Temporal Gyrus DLPFC: Dorsolateral Prefrontal Cortex IFC: Inferior Frontal Cortex. Evidence Grading: +++: consistent benefit (moderate/high-confidence meta-analysis, guidelines, or ≥2 low-bias RCTs with concordant results); ++: probable benefit (low-confidence review or ≥2 RCTs with generally consistent results); +: preliminary support (single RCT or non-randomized evidence); +/−: mixed or insufficient evidence; −: no benefit in controlled evidence; blank: no eligible data.

#### Symptomatic therapy for cardinal motor symptoms

Multiple systematic reviews focusing on RCTs^10-12^ including one of moderate quality, have reported positive effects of rTMS on motor symptoms in Parkinson’s disease. Similar conclusions have been drawn by broader reviews that also including open-label studies.^13-18^ These reviews led to identify high-frequency (HF)-rTMS (5–50 Hz) of bilateral regions of the primary motor cortex (M1) as probably effective in reducing motor symptoms of Parkinson’s disease.^8^ Effect size was consistently larger in studies based on repeated sessions of HF rTMS of M1 rather than on low-frequency (LF) rTMS (1 Hz) of M1, which usually provide no clinical benefit in patients with Parkinson’s disease.^19,20^

Among the various RCTs published in this domain, several studies showed prolonged clinical benefit on motor symptoms beyond the time of stimulation. For example, Khedr *et al.*^21^ (*n* = 30) showed a significant improvement in the UPDRS-III motor score after 10 sessions of 20 Hz rTMS delivered to bilateral M1 regions (39.7 ± 14.5) when compared to baseline and sham stimulation (61.9 ± 13.2 and 61.0 ± 17.5, respectively), which was maintained at subsequent assessments over five monthly maintenance sessions (45.8 ± 13.1). Similar results were found in another trial by Makkos *et al.*^22^ (*n* = 44) based on 10 sessions of 5 Hz rTMS delivered to bilateral M1 regions, with a significant reduction in UPDRS-III motor score, associated with an improvement in quality of life, observed at the end of treatment for active but not sham rTMS and sustained 30 days after the last rTMS session. Sustained effects were also described by Chung *et al.*^23^ (*n* = 51) who performed 3 weeks of daily rTMS sessions delivered to M1 at either 1 or 25 Hz, combined with treadmill training: the decrease in UPDRS-III motor score produced by M1 rTMS, regardless of stimulation frequency (1 Hz: 21.9 ± 7.8; 25 Hz: 22.5 ± 10.1), was still significant 3 months after the treatment period compared to baseline scores and treadmill training with sham rTMS (27.9 ± 10.5, 27.1 ± 9.6, 27.3 ± 8.0).

Overall, improvement in global motor symptoms appears to be driven mainly by high-frequency bihemispheric M1 stimulation, delivered sequentially over both hemispheres with a figure-of-eight (F8) focal coil^24-28^ or a large double-cone coil^23^ for medial M1 region.^23-28^ The exact subregion varied across studies: some targeted more medial M1/leg representations, whereas others used hand motor areas. Trials targeting premotor medial cortical regions, such as the supplementary motor area (SMA), also found positive results on UPDRS-III motor score, which were maintained 4 weeks^29^ to 12 weeks^30^ after the end of intervention. However, regarding the SMA target, unlike the M1 target, a motor benefit has been reported using either LF (1 Hz) or HF (5 Hz) rTMS,^30-33^ or even using continuous theta burst stimulation (cTBS),^34,35^ revealing the impossibility of concluding on an optimal protocol to be designed to modulate this cortical region as effectively as possible. Some evidence suggests that bihemispheric rTMS targeting both M1 and DLPFC may improve motor symptoms. In one sham-controlled study, 25 Hz HF-rTMS was delivered with an F8 coil to both regions sequentially during each session over several days.^36^ Although UPDRS-III scores were not significantly reduced, improvements in bradykinesia and gait were noted. These effects were not replicated when the team used iTBS instead of HF-rTMS.^37^ Some studies used a large H-coil to stimulate both M1 and DLPFC, typically applying LF (1 Hz) rTMS to M1 and HF (10 Hz) to the prefrontal area. Four of six studies reviewed reported significant motor improvements on the UPDRS-III score.^38^

Current data do not support a specific effect of any rTMS protocol on tremor, bradykinesia or rigidity in Parkinson’s disease. However, the impact on gait and postural stability appears to be smaller,^10-12,17,18^ as detailed below. Interestingly, most trials included Parkinson’s disease patients with varied phenotypes and disease durations, suggesting rTMS may be effective across different stages. However, its effects may vary by phenotype and symptom profile, likely reflecting differences in motor cortical excitability.^39-41^

#### Treatment of motor fluctuations

Our scoping review found no rTMS studies specifically targeting motor fluctuations. Some trials using diaries^42^ showed no effect, though these were not designed for that purpose. Notably, HF rTMS over M1 consistently improves ON motor symptoms but has less impact during OFF states.^12^ Dedicated trials are needed to assess effects on total ON/OFF time.

#### Treatment of levodopa-induced dyskinesias

We have identified 14 original studies,^31,42-54^ representing 16 independent cohorts, where effects of rTMS for levodopa-induced dyskinesias were tested. Among 14 studies, 11 used randomized parallel or crossover designs and were eligible for RoB 2 assessment (Supplementary Fig. 1). Most raised some concerns, mainly due to incomplete reporting of randomization/allocation procedures, possible carry-over effects and lack of prespecified analysis plans. Study details on Supplementary Table 2.

Acute effects of a single session of rTMS in levodopa-induced dyskinesias was tested in nine cohorts.^42,44-49,53,54^ Assessments were performed using clinician-rated tools, in most cases blinded ratings of patient videos using the Abnormal Involuntary Movement Scale (AIMS) or equivalent. Dyskinesias diaries or patient-reported dyskinesia assessment tools (UPDRS-IV, 26-item Parkinson Disease Dyskinesia Scale, Lang-Fahn Activities of Daily Living Dyskinesia Scale) were used in five of the cohorts where multiple sessions were performed. A sixth study used a patient-reported Visual Analogue Scale of dyskinesias.

In Parkinson’s disease patients with dyskinesias, a single rTMS session applied before or after a levodopa dose reduced dyskinesia severity when delivered at 1 Hz to bilateral SMA, compared to 5 Hz or sham (*n* = 8,^54^  *n* = 10^53^). This was confirmed in an RCT (*n* = 17) showing significant reduction in dyskinesias after 10 sessions of 1 Hz rTMS over bilateral SMA, assessed by AIMS and VAS. Similar effects were seen in two studies (*n* = 10,^31^  *n* = 6^52^) using 8–10 sessions of 1 Hz rTMS targeting M1.

A more anterior region (pre-SMA) was the target of two studies based on single session of LF (1 Hz) rTMS. The first was negative (*n* = 9),^44^ but the second reported positive results (*n* = 17), attributed to a higher number of pulses (1800 versus 1000) and stimulation intensity (100% RMT versus 90% RMT).^45^

Positive results also emerged with cTBS protocols delivered to the cerebellum.^42,50^ A single-arm study of the effects of a single session of unilateral cerebellar cTBS found a reduction in dyskinesias (*n* = 11), with similar results in a sham-controlled study with a similar stimulation protocol (*n* = 20). An RCT with 10 sessions of bilateral cerebellar cTBS (*n* = 20) led to a reduction in clinician-rated dyskinesias, that was maintained at least 2 weeks after the end of treatment. This was also verified in patient diaries, revealing a decrease in the number of hours spent in ON with dyskinesias, also maintained 1 week after end of rTMS and that was not due to a reduction of global quality or the time spent in ON. A similar reduction in clinician-assessed dyskinesias was also seen in a crossover study after five sessions of bilateral cerebellar cTBS (*n* = 8).^50^ Finally, a study showed that the antidyskinetic effect produced by 10 sessions of bilateral cerebellar cTBS paralleled sensorimotor cortical plasticity assessed by a paired-associative stimulation technique.^55^

In addition to (pre)motor cortical regions and the cerebellum, two other targets were tested for potential antidyskinetic effects, namely the DLPFC and the Inferior frontal cortex (IFC). In one study, five sessions of HF (10 Hz) rTMS of the left DLPFC tended to improve levodopa-induced dyskinesias in four patients with advanced Parkinson’s disease.^43^ In two other studies, a single session of cTBS delivered to the IFC led to a reduction in AIMS score (*n* = 10,^47^  *n* = 11^49^), a result not seen with iTBS protocols (*n* = 11).^49^

#### Prevention of motor complications

To our knowledge, no study has specifically examined delaying motor complications. Given that HF rTMS of M1 improves global motor symptoms, it is plausible that concurrent rTMS could reduce levodopa requirements and lower the risk of motor complications. This parallels findings from the CALM-PD^56^ study, where add-on pramipexole resulted in lower levodopa doses after 4 years (434 mg/day versus 702 mg/day) and reduced rates of dyskinesias (24.5% versus 54.0%) and wearing off (47.0% versus 62.7%).

#### Treatment of gait dysfunction and freezing of gait

The role of rTMS on gait dysfunction, especially freezing of gait (FOG) has been a major topic of research and multiple systematic reviews have addressed this topic.^57-62^ Outcomes have included Timed-Up and Go (TUG) tests, objective gait assessment (e.g. time or speed of walking a specific distance), the Freezing of Gait Questionnaire (FOG-Q) or the UPDRS gait parameters.

A meta-analysis including 16 RCTs with a total of 408 patients with Parkinson’s disease and FOG identified M1 and SMA as relevant stimulation targets for FOG.^62^ There was no support for isolated DLPFC stimulation,^63^ with results produced by targeting DLPFC weaker than those produced by stimulation of M1.^64^ Positive results have been observed by applying LF (1 Hz) rTMS over leg M1^14,43^ or HF (10–25 Hz) rTMS over leg M1^14,42,50^ or SMA,^17^ consistent across multiple metrics (FOG-Q, Walking time or TUG). Thus, for example, 10 sessions of HF (10 Hz) rTMS delivered bilaterally to M1 (*n* = 44^65^) or to SMA (*n* = 30^65^ or *n* = 28^65^) were found to improve FOG-Q score up to 1 month after the end of treatment (active: 13.41 to 10.55 versus sham 13.55 to 13.55^65^; active: 16.04 to 13.91 versus sham 16.00 to 16.40^17^; active: 14.79 to 12.68 versus sham 14.20 to 13.87^66^). In all of these trials,^47,50,67^ a global motor improvement was also observed on the UPDRS-III score. SMA stimulation was generally delivered as a mid-line, bihemispheric target and DLPFC was mostly unilateral left-sided.

#### Treatment of dysarthria and dysphagia

Regarding the impact of rTMS protocols on speech impairment, we identified five original studies^68-72^ in Parkinson’s disease (Supplementary Table 3) raising some concerns on RoB 2 (Supplementary Fig. 2), mainly due to uncertainty around blinding or sham adequacy, potential carry-over effects in crossover designs and lack of prespecified analysis plans. Ten or more rTMS sessions were performed at LF (1 Hz) over the right superior temporal gyrus (STG),^71^ a node of auditory and phonological processing, or at HF (5–15 Hz) over the laryngeal M1 representation^70^ or the left DLPFC.^55^ Heterogeneous assessment metrics were used: acoustic analysis, standardized clinical assessment of dysarthria and voice-related quality of life tools. A positive effect on various phonetic dimensions (articulation, prosody and intelligibility) was produced by 10 sessions of LF (1 Hz) rTMS of the right STG, sustained for up to 8 weeks after the end of the treatment.^71^ In a previous small crossover study (*n* = 16), the same authors showed acute beneficial effects on speech of LF rTMS of the right STG, but not of HF rTMS, nor M1 stimulation.^69^ Another trial based on HF (5 Hz) rTMS of the laryngeal M1 representation combined with speech and language therapy (SLT) (*n* = 20)^70^ found no significant impact of the rTMS procedure. In contrast, other studies showed that HF (5–10 Hz) rTMS of the laryngeal M1 representation (*n* = 8)^72^ and (*n* = 12)^68^ could improve voice quality and intensity.

Regarding dysphagia, in one RCT^21^ (*n* = 33) 10 sessions of HF (20 Hz) rTMS targeting the hand region of M1 bilaterally were found to produce an improvement in the dysphagia handicap index and in transit time as assessed by video-fluoroscopy. Improvements were maintained after 3 months with monthly maintenance sessions (five sessions/month). A single-arm study (*n* = 38) also described the improvement in dysphagia after 10 sessions of HF (10 Hz) rTMS delivered unilaterally to an unspecified location of M1^73^ (details in Supplementary Table 4 and Supplementary Fig. 3).

### Non-motor symptoms

Data presented here is summarized in Table 2.

**Table 2:** Summary of rTMS effects on non-motor symptoms in Parkinson’s disease

Symptom	M1	SMA	DLPFC (L)	DLPFC (R)	Insula	Parietal
Depression	+ (HF/LF)		+++ (HF)	+ (LF)		
Anxiety	+/− (HF)				− (HF)	
Apathy	− (HF)	− (LF/HF)	− (HF)	+ (HF)		
Sleep disturbance				++ (LF)		+ (HF)
Fatigue						
Pain	+ (HF)				+/− (HF)	
Cognition (MCI)			++ (HF), ++ (iTBS)	+ (HF)		
Dementia	+ (HF)					
Impulse control			+/− (LF)			
Psychosis						
Behavioural changes						
Autonomic symptoms	+/− (LF)*		+/− (HF)*	+/− (HF)*		

This table summarizes the current evidence regarding the effects of repetitive transcranial magnetic stimulation (rTMS) on non-motor symptoms in Parkinson’s disease, detailing targeted brain regions, stimulation frequencies and levels of evidence. The table categorizes symptoms addressed by rTMS, and the strength and quality of available evidence for each of them. Protocol types: HF: High-Frequency rTMS (≥5 Hz, commonly 10–20 Hz) LF: Low-Frequency rTMS (typically 1 Hz) cTBS: Continuous Theta Burst Stimulation, iTBS: Intermittent Theta Burst Stimulation Targets: M1: Primary Motor Cortex SMA: Supplementary Motor Area, DLPFC: Dorsolateral Prefrontal Cortex (R): Right, (L): Left, MCI: Mild Cognitive Impairment Evidence Grading: +++: consistent benefit (moderate/high-confidence meta-analysis, guidelines, or ≥2 low-bias RCTs with concordant results); ++: probable benefit (low-confidence review or ≥2 RCTs with generally consistent results); +: preliminary support (single RCT or non-randomized evidence); +/−: mixed or insufficient evidence; −: no benefit in controlled evidence; blank: no eligible data.

#### Treatment of anxiety, depression and apathy

Evidence-based guidelines support the probable antidepressant efficacy of HF-rTMS of the left DLPFC in Parkinson’s disease,^8^ based on multiple clinical trials and systematic reviews.^12^ Its effect appears similar or slightly superior to selective serotonin reuptake inhibitors (e.g. fluoxetine),^74,75^ with one study showing comparable benefits to pramipexole and slightly lower efficacy than escitalopram after 4 weeks.^76^ One trial also found that LF (1 Hz) rTMS of the right DLPFC reduced depressive symptoms in Parkinson’s disease,^77^ consistent with findings in the general depressed population. Additionally, a few studies have reported antidepressant effects following M1 stimulation in Parkinson’s disease.^22,28,78^

There is less evidence for other psychiatric symptoms. Regarding anxiety, no specific study used it as a main endpoint. We identified two trials^78,79^ addressing anxious symptoms presented by Parkinson’s disease patients as a secondary endpoint of M1 rTMS protocols showed outcomes with very low effect size. A third trial focusing on insular stimulation for pain treatment found no effect in anxiety.^80^

For apathy, four rTMS studies have been identified in Parkinson’s disease patients using the apathy evaluation scale, Lille apathy rating scale, Starkstein apathy scale and the non-motor symptoms questionnaire apathy subscore. Two studies evaluated short-term effects (<2 days) and two others long-term effects (20 weeks and 6 months). Only one study, based on HF (10 Hz) rTMS of the right DLPFC found a significant reduction of apathy, with a positive impact on emotional processing more marked in apathetic than non-apathetic Parkinson’s disease patients.^81^ The remaining studies (HF (10 Hz) of left DLPFC and Bilateral M1,^28^ HF (5 Hz) of left DLPFC,^82^ or LF (1 Hz) or HF (10 Hz) of bilateral SMA^30^) did not show beneficial effects.

#### Treatment of sleep disorders and fatigue

We identified six trials (Supplementary Table 5) where sleep metrics were assessed.^77,83-86^ Overall, they were judged as raising some concerns of bias, mainly because of limited information on blinding procedures, and lack of clearly prespecified analysis plans (Supplementary Fig. 4). Concerns were lower for studies using objective PSG or actigraphy outcomes. Three trials based on LF (1 Hz) rTMS of the right DLPFC found beneficial effects. One study described significant improvement on polysomnography up to 3 months after the trial (*n* = 67).^87^ Daytime sleepiness improved for up to 1 month as assessed by Epworth sleepiness scale (*n* = 25)^85^ in a second trial. The third study found significant results, albeit below thresholds for clinical significance, on the Pittsburgh sleep quality index (active: 9.63–7.30; sham: 7.57–8.36; *n* = 33^77^). Two other trials used HF (5 Hz,^83^  *n* = 13; 20 Hz,^86^  *n* = 24) rTMS of bilateral parietal cortices and showed positive effects in objective assessments (actigraphy and polysomnography) and in the Parkinson’s sleep scale. In a sixth trial, LF (1 Hz) rTMS delivered over the vertex did not produce relevant subjective or objective effects on sleep.^84^ We did not find data regarding the treatment of specific sleep disorders (e.g. REM-Sleep Behaviour Disorder) with rTMS.

Although fatigue in Parkinson’s disease has been associated with changes in cortical excitability,^88^ we also did not find rTMS trials specifically focusing on the treatment of fatigue in Parkinson’s disease patients.

#### Treatment of pain

In the context of Parkinson’s disease, there are two main rTMS trials focusing on the analgesic effects. In a 8-week sham-controlled trial (*n* = 25) applying HF (10 Hz) rTMS to posterior–superior insula results were globally negative, although a *post hoc* analysis suggested an improvement in patients with nociceptive pain.^80^ The second trial, where five sessions of HF (20 Hz) rTMS were applied to M1 contralateral to the side of pain, a significant reduction of the intensity of pain (of ∼3 points on a 0–10 numeric rating scale) was observed for 4 weeks beyond the period of stimulation.^78^ In addition, a case report described that LF rTMS delivered to M1 but not to SMA, could have an impact on painful dystonia associated with Parkinson’s disease.^89^

#### Treatment of cognitive symptoms and dementia

Similarly to a recent systematic review,^90^ we have identified 22 papers addressing the role of rTMS in cognition in Parkinson’s disease patients, with most trials using Montreal cognitive assessment (MoCA) or mini mental state examination (MMSE) questionnaires as the main assessment tools. These studies would mostly raise some concerns on risk of bias, driven by unclear allocation concealment and outcome-assessor blinding, despite generally low risk for missing outcome data and no clear evidence of selective reporting. The rTMS protocols targeted the DLPFC in all cases, on the left hemisphere (10 trials), on the right hemisphere (two trials), or bilaterally (four trials). A network metanalysis^90^ revealed that HF rTMS of the left DLPFC, with 4-week treatment duration, may enhance cognitive function with effects that could be maintained up to 1 month after the last rTMS session. At least six articles^91-94^ showed that HF (5–10 Hz) or iTBS of the left DLPFC applied to patients with Parkinson’s disease and mild cognitive impairment can improve visuospatial functions, verbal fluency, executive functions or delayed recall metrics. A sham-controlled trial in patients with Parkinson’s disease and dementia (*n* = 36)^95^ found that 10 sessions of HF (20 Hz) delivered bilaterally to M1 stimulation, followed by monthly maintenance sessions, led to a small but significant improvement of both MoCA and MMSE scores. This was accompanied by an improvement in activities of daily living as well as in motor symptoms on the UPDRS-III score.^95^

#### Treatment of impulse control disorders

A small (*n* = 4) proof of concept study^96^ found that a single session of LF (1 Hz) rTMS delivered to the DLPFC (particularly on the right hemisphere) was able to transiently reduce severe punding for 12 h.

#### Treatment of hallucinations, delusions and other psychotic symptoms

Beyond one single case report of rTMS applied to the occipital lobe to treat visual hallucinations in an 82-year-old Parkinson’s disease patient,^97^ we did not identify studies addressing the impact of rTMS on psychotic symptoms in Parkinson’s disease.

#### Treatment of behavioural changes

Most behavioural disturbances in Parkinson’s disease result from combinations of the symptoms described above. We found no studies specifically addressing this dimension.

#### Treatment of urinary and other autonomic symptoms

An open-label study of 8 patients with advanced Parkinson’s disease^98^ and lower urinary tract symptoms found that 10 sessions of LF (1 Hz) rTMS over M1 significantly improved self-reported symptoms and bladder capacity, with effects lasting up to 2 weeks. Another observational study (*n* = 45)^99^ using bihemispheric stimulation with LF (1 Hz) rTMS over M1 followed by HF (10 Hz) rTMS over DLPFC via an H-coil reported broad clinical improvements, including autonomic symptoms, based on self-rated scales.

## Discussion

We reviewed evidence for rTMS as a potential therapeutic tool for Parkinson’s disease within a long-term management framework. Across domains such as motor symptoms and depression, multiple RCTs and systematic reviews suggest clinical benefit, but the field remains influenced by small trials, short stimulation protocols, heterogeneous targets, inconsistent outcome reporting and variable methodological quality, all of which limit the strength of conclusions. At the same time, some of the most disabling features of Parkinson’s disease remain insufficiently treated, with only a handful of pharmacological options as clinically useful in these domains. In this context, the repeated observation of positive signals with rTMS is encouraging, and underscores the need for well-powered, circuit-informed trials that refine stimulation protocols, improve patient selection and standardize outcome measures. A major unresolved issue across the Parkinson’s disease rTMS literature is dosage: although repeated multi-session protocols generally outperform very brief interventions, heterogeneity in session number, pulse dose, treatment spacing and maintenance schedules still prevents firm symptom-specific recommendations. In addition, although most studies included medicated patients, medication state was often poorly reported and insufficiently controlled, particularly with respect to ON/OFF status and the timing of treatment sessions and outcome assessments relative to dopaminergic medication, introducing an additional source of variability and interpretative uncertainty. Table 3 outlines the domains where this agenda is most urgent, and where rTMS could potentially complement established therapies. While it is useful to contextualize rTMS findings alongside existing pharmacological options, such comparisons remain indirect and should be interpreted cautiously given the heterogeneity of trial designs, patient populations, disease stage, outcome measures and short follow-up periods. Moreover, these contextual comparisons should not be interpreted as equivalent to the formal evidence-grading processes used by guideline committees, which rely on structured assessments of methodological quality, consistency and clinical applicability. In the sections that follow, we critically appraise rTMS across symptomatic domains and situate its effects alongside current standard therapies, while explicitly acknowledging that cross-trial comparisons are contextual and cannot establish therapeutic equivalence. Our intent is not to reclassify treatments against existing guideline categories, but to highlight where recurrent rTMS signals may justify more definitive trials within domains of persistent unmet need.

**Table 3:** Potential research priorities for rTMS in Parkinson’s disease

Research topic	Evidence and needs
Motor symptoms	Evidence for high-frequency M1 stimulation in motor improvement. Optimal protocols and maintenance strategies (including rehabilitation) remain unclear. Lack of data regarding dissociable symptom effects (e.g. tremor versus bradykinesia).
Motor fluctuations	No dedicated studies on motor fluctuations. Potential indirect benefit through motor improvement.
Dyskinesias	Preliminary evidence suggests LF rTMS to SMA and cTBS to cerebellum may reduce dyskinesias. Lack of multi-session studies
Prevention of motor complications	No studies have directly tested rTMS for delaying motor complications or dyskinesias. Given that HF-rTMS of M1 improves motor symptoms, it may reduce levodopa dose requirements, potentially delaying dyskinesias and motor fluctuations.
Freezing of gait	Meta-analyses suggest positive effects, especially with SMA/M1 stimulation. Dissociation between global motor improvement and gait-specific effects is unproven.
Speech and dysphagia	Preliminary data supports right STG stimulation for dysarthria and M1 stimulation for dysphagia and dysarthria. Larger trials are needed with combined strategies including speech and language therapy.
Depression	Well-established efficacy of HF-rTMS of left DLPFC in major depression, supported by Parkinson’s disease-specific trials. Optimization of protocols in Parkinson’s disease needed, including assessment of accelerated protocols.
Anxiety	Limited trials in Parkinson’s disease. General anxiety disorder studies suggest DLPFC as a promising target
Apathy	Only one positive trial (HF-rTMS of right DLPFC) with depression as confounder. Heterogeneity in apathy mechanisms requires refined patient stratification.
Cognitive symptoms	DLPFC stimulation shows cognitive benefits in Parkinson’s disease patients and in specific symptoms. Explore long-term effects and differential impact on specific cognitive domains. Understand mechanistic effects
Impulse control disorders	Direct targeting of compulsive behaviour circuits needs investigation. Indirect benefits possible through reduced medication, but targeted studies are needed
Pain	HF-rTMS of M1 has clear effects in neuropathic pain in other clinical contexts. Lack of studies in Parkinson’s disease. Pain in Parkinson’s disease has multiple sources and pathophysiological mechanisms. Stratified research is necessary to understand potential benefit in each specific conditions of comorbid pain syndrome associated with Parkinson’s disease.
Sleep disorders	Positive results in sleep quality with various rTMS protocols and cortical targets. Sleep disorder-specific trials are lacking.
Psychotic symptoms	No studies in Parkinson’s disease. Psychosis in Parkinson’s disease is strongly linked to dopaminergic therapy. If rTMS reduces the need for higher doses, it could help prevent psychotic symptoms. No trials have tested direct neuromodulation for psychosis prevention in Parkinson’s disease.
Urinary and autonomic symptoms	Preliminary evidence for various rTMS protocols in improving urinary function of Parkinson’s disease patients. Benefit of rTMS on Parkinson’s disease-associated autonomic symptoms remain speculative and require further controlled trials.

This table outlines key areas where further research is needed to assess the therapeutic potential of rTMS in Parkinson’s disease. For each symptom or complication, relevant arguments supporting investigation are provided, highlighting existing evidence gaps and possible mechanisms of action.

### Motor symptoms

#### Cardinal motor symptoms

High-quality evidence supports the use of rTMS to improve motor symptoms in Parkinson’s disease. However, critical uncertainties remain. It is unclear whether stimulation should be performed in the ON or OFF state, and whether therapeutic effects differ across these conditions. Few studies focus on specific motor symptoms or Parkinson’s disease subtypes, and the optimal design of maintenance protocols remains unresolved. Nevertheless, systematic reviews and multiple RCTs consistently identify high-frequency bilateral rTMS of M1 as the most effective approach, with effects sustained for weeks to months when protocols are repeated. Individual studies have shown absolute improvements in UPDRS-III ranging from 4 to 16 points, often coupled with better quality of life. Stimulation of the SMA^12,13,67^ has also yielded durable motor benefits, though optimal frequency remains less defined with cerebellum^16,100^ and multisite stimulation^28^ bringing potential additional benefit.

When contextualized against standard therapies, rTMS appears modest if judged by global meta-analyses, which often pool together short, unilateral or low-frequency protocols with effective protocols (SMD ≈ −0.46).^101^ Yet, if attention is restricted to bilateral, high-frequency, multi-session stimulation of M1 or SMA, the picture shifts. Under these conditions, rTMS produces 5–10 point improvements in UPDRS-III, corresponding to moderate-to-large effect sizes (SMD −0.6 to −0.8) and consistently exceeding the MCID threshold of 3–5 points.^102^ By comparison, acute levodopa produces ∼9–12 point gains.^103,104^ Adjunctive pharmacotherapies yield more variable results: pramipexole improves UPDRS-III by ∼6 points (SMD ≈ −2.6), and rasagiline by ∼4 points (SMD ≈ −2.5) in early Parkinson’s disease trials.^105-107^ However, other meta-analyses on rasagiline report smaller effects (SMD ≈ −0.3),^108^ underscoring the inconsistency of effect size estimates across the literature, likely driven by heterogeneity in symptoms, trial design and disease stage. For this reason, Absolute UPDRS changes (e.g. 4–6 points for pramipexole, 3–4 points for rasagiline) remain a crucial benchmark to provide clinically intuitive comparisons.^105-108^ While rTMS does not approach the magnitude of benefit typically seen with levodopa, optimized protocols yield effect estimates that appear clinically relevant when viewed alongside some adjunctive pharmacological therapies; however, these comparisons are indirect and should not be interpreted as evidence of therapeutic equivalence. Although several RCTs report persistence of motor benefits beyond the stimulation period, with follow-up durations ranging from 30 days to 3 months and, in one study, across monthly maintenance sessions, the evidence remains too limited and heterogeneous to support strong conclusions regarding long-term therapeutic benefit. Defining the duration of therapeutic effects, the need for maintenance stimulation and how protocols can be integrated into regular clinical care therefore remains a key research priority.

#### Unmet needs in motor symptoms: Dyskinesias, gait, posture, dysarthria and dysphagia

Several of the most disabling motor features of Parkinson’s disease remain poorly treated by available therapies, creating a space where rTMS could have a transformative impact. FOG is perhaps the clearest example: the 2018 MDS guidelines list only rivastigmine as ‘possibly useful’ for gait and balance, based on a single trial, while evidence for other pharmacological agents was judged.^2^ By contrast, rTMS has already been tested in more than a dozen randomized controlled trials, with meta-analyses of 16 RCTs^62^ (*n* = 408) showing reproducible, small-to-moderate improvements in FOG-Q (SMD ≈ 0.28) and consistent gains in Timed-Up-and-Go and walking speed. Importantly, optimized bilateral high-frequency protocols over M1 or SMA yield 2–3 point FOG-Q reductions sustained for weeks, coupled with parallel UPDRS-III improvements. Although direct comparative conclusions cannot be drawn, these findings suggest that rTMS deserves consideration alongside the limited pharmacological options currently available for FOG.

For dyskinesias,^12,15,16^ the current evidence for rTMS remains limited but mechanistically grounded. Early rTMS studies targeting SMA and cerebellar circuits show promising reductions in levodopa-induced dyskinesias, consistent with pathophysiological models of abnormal basal ganglia–cerebellar synchronization. The 2018 MDS guidelines underscore how narrow the current therapeutic landscape is: beyond amantadine and clozapine, few pharmacological options are considered clinically useful, leaving a substantial gap outside surgery or infusion therapies.^2^ In this context, rTMS stands out as a non-invasive, scalable candidate. Future trials should use standardized outcome measures and assess whether rTMS can not only reduce dyskinesias but also mitigate fluctuations, lower levodopa burden and contribute to safer, circuit-based management of treatment complications in Parkinson’s disease.

Dysarthria and dysphagia further highlight the unmet need. These symptoms undermine communication, nutrition and quality of life,^109,110^ yet beyond speech–language therapy, effective options are scarce. Pilot rTMS studies report encouraging gains: low-frequency stimulation over the superior temporal gyrus has improved dysarthria, while stimulation of orofacial M1 and adjacent regions shows potential for dysphagia. A recent meta-analysis in post-stroke dysphagia (10 studies, 246 patients) also revealed that M1 rTMS significantly improved swallowing function (SMD = −0.76),^111^ improved autonomy and reduced aspiration risks. If paired with structured rehabilitation, rTMS in Parkinson’s disease could add a circuit-specific intervention to a domain otherwise left to supportive care.

Beyond addressing these domains individually, there is a compelling rationale that adjunctive rTMS might smooth motor symptoms, reduce reliance on escalating dopaminergic doses and ultimately mitigate the risk of motor fluctuations and dyskinesias. Testing this hypothesis requires longer, mechanistically informed trials that integrate rTMS with dopaminergic therapy and monitor fluctuations, levodopa dosage and dyskinesia severity alongside clinical outcomes.

Taken together, FOG, dyskinesia and bulbar dysfunction stand as major unmet motor needs where pharmacological options are minimal and surgical solutions are limited to specific cases. rTMS already offers reproducible benefits in some of these domains, and with optimized protocols and circuit-informed trial design, it could evolve into a non-invasive, scalable therapy that meaningfully expands the treatment arsenal for Parkinson’s disease.

### Non-motor symptoms

#### Psychiatric symptoms in Parkinson’s disease: where rTMS can complement standard care

The 2019 MDS non-motor guidelines already recognize rTMS as ‘possibly useful’ for depression in Parkinson’s disease,^112^ though they caution that effects appear short-term and would require repeated treatment courses. This cautious appraisal reflects the small size and limited duration of Parkinson’s disease-specific trials. However, broader psychiatric experience points to a more optimistic interpretation. In major depression, rTMS is an established therapy with regulatory approval,^8^ and large multicentre real-world evidence studies involving more than 500 patients have demonstrated effectiveness even in older adults^113^: interesting, in this population, clinical response may take longer to emerge. These findings suggest that the guideline emphasis on ‘short-term’ effects may underestimate the true potential of rTMS if longer courses or maintenance protocols are used (The pharmacological treatment of depression usually involves at least 6–12 months of maintenance.). Importantly, accelerated iTBS paradigms,^114,115^ which are now showing rapid and robust antidepressant responses in psychiatry, could be particularly relevant in Parkinson’s disease. They not only reduce treatment burden in patients with mobility limitations but may also fit better with the clinical reality of fluctuating patients, where faster onset of benefit and shorter treatment windows could prove decisive. Compared with current standard options: venlafaxine and pramipexole (‘clinically useful’), SSRIs/SNRIs and CBT (‘possibly useful’) and ECT for refractory cases, rTMS offers a non-sedating, non-systemic option that avoids anticholinergic and orthostatic side effects and drug–drug interactions common in dopaminergic regimens.

For Parkinson’s disease-related anxiety, the guidelines judged evidence for all interventions as ‘insufficient’, reflecting modest and inconsistent results with medications and CBT. DLPFC rTMS is effective for generalized anxiety^116^ outside Parkinson’s disease and may extend to Parkinson’s disease (especially in patients with comorbid depression) but targeted trials are still needed. Apathy likewise remains insufficiently treated: rivastigmine and piribedil after STN-DBS are rated ‘possibly useful’, but no consistently effective pharmacological option exists. Preliminary evidence that right-DLPFC rTMS can improve emotional processing in apathetic Parkinson’s disease patients points to a tractable network target,^81^ though this requires validation in larger, stratified cohorts. Network mapping tools may provide network guidance in this specific topic.

A major challenge is disentangling overlapping from domain-specific effects. Improvements in mood following M1 stimulation may partly reflect better motor function, while motivational gains may overlap with relief of depressive affect. Conversely, true apathy syndromes may persist despite successful antidepressant treatment.^117^ For rTMS to take a clearer place in Parkinson’s disease care, future studies must explicitly parse what is shared across depression, apathy and motor symptoms and what is specific—using multidimensional outcome measures, stratified patient populations and network-informed stimulation strategies.

About half of Parkinson’s disease rTMS studies focusing on sleep report improvements, usually with low-frequency right-DLPFC stimulation, but protocols remain inconsistent. The guidelines list rotigotine as ‘possibly useful’ for insomnia, and rasagiline as ‘possibly useful’ for fatigue, yet evidence across both domains is weak. No rTMS trials have directly addressed fatigue in Parkinson’s disease, although evidence in other conditions suggests left-DLPFC stimulation may be beneficial.^118,119^ For impulse control disorders dopaminergic dose reduction remains the mainstay.^120^ The established efficacy of rTMS in obsessive–compulsive disorder makes fronto-striatal neuromodulation a plausible future option in Parkinson’s disease, though no dedicated trials exist. Importantly, many of these non-motor complications (including sleep disruption and ICDs) are aggravated by dopaminergic therapy. This raises the possibility that adjunctive rTMS could indirectly mitigate such complications by stabilizing motor function and reducing reliance on escalating dopaminergic regimens.

In psychiatric complications of Parkinson’s disease, where available treatments are effective but constrained by side effects, drug–drug interactions or incomplete efficacy, rTMS meets the bar of a ‘possibly useful’ intervention for depression and shows actionable signals for anxiety, apathy and sleep. The next step is not only larger, adequately powered trials, but also designs that can distinguish shared from domain-specific effects, ensuring that observed improvements are both clinically meaningful and aligned with the complex neuropsychiatric burden of Parkinson’s disease.

#### Pain

Pain in Parkinson’s disease is heterogeneous, ranging from nociceptive to nociplastic and neuropathic mechanisms, and remains an unmet therapeutic need.^121^ The MDS evidence-based guidelines highlight how narrow the therapeutic landscape is: rotigotine is rated ‘investigational’ and oxycodone/naloxone prolonged-release only ‘possibly useful, largely on the basis of efficacy outside Parkinson’s disease.^112^ In contrast, rTMS has well-established analgesic effects in non-Parkinson’s disease conditions, with HF-M1 stimulation recognized for neuropathic pain^8^ and HF-DLPFC protocols for nociplastic syndromes such as fibromyalgia.^8^ Evidence in Parkinson’s disease is still scarce, but available studies suggest that phenotype-specific targeting could be clinically meaningful.^78^ Future trials must separate direct analgesic effects from indirect motor gains, stratify patients according to pain subtype and test mechanism-matched targets. If effective, rTMS would represent a non-opioid, non-sedating and scalable option that may be clinically relevant alongside guideline-endorsed pharmacological alternatives.

#### Cognitive impairment

Cognitive dysfunction in Parkinson’s disease remains one of the most stubborn non-motor challenges, with few effective treatments. According to the 2019 MDS non-motor guidelines, rivastigmine is rated ‘clinically useful’ for Parkinson’s disease dementia, while donepezil and galantamine are considered ‘possibly useful’. All other options garnered only ‘insufficient evidence’.^112^ Into this sparse landscape, rTMS offers an intriguing, potentially scalable alternative. In Parkinson’s disease, most trials have used HF-rTMS or iTBS over the left dorsolateral prefrontal cortex (DLPFC), yielding modest but meaningful gains in global cognition that can persist for up to 1 month.^90^ However, part of this improvement may stem from indirect effects on mood or motor performance rather than cognition *per se*. A meta-analysis of cholinesterase inhibitors in Parkinson’s dementia showed small effect sizes for MMSE (1–2 points benefit versus placebo) and ADAS-Cog, indicating that gains from existing drugs, while significant, are modest.^122^ The signal being obtained from rTMS studies is at least in the same order, however, heterogeneity across targets and protocols limit interpretation of the potential.

Evidence from non-Parkinson’s disease context may inform Parkinson’s disease research and is cited here as supportive context for target selection and biological plausibility, rather than as direct evidence for efficacy in Parkinson’s disease. A systematic review found a medium-to-large overall effect (SMD ≈ 0.77) favouring active rTMS over sham in elderly cognitive disorders—including early Parkinson’s disease cohorts.^123,124^ In mild cognitive impairment (not Parkinson’s disease specific), pooled rTMS studies targeting DLPFC achieved an SMD = 0.83 (95% CI 0.44–1.22), and memory function improved even more strongly (SMD = 0.73).^125^ In Alzheimer’s disease, network-based protocols like precuneus stimulation over 52 weeks (targeting default mode network hubs) notably slowed cognitive and functional decline with a significant ∼5 points group difference in the ADAS-Cog11 and a non-significant (*P* = 0.051) ∼2 points in the MMSE.^126,127^

Taken together, indirect comparisons suggest that rTMS may produce cognitive effects of potential clinical relevance in Parkinson’s disease, but current evidence remains too heterogeneous and confounded by mood, motor and protocol differences to support firm efficacy conclusions.

#### Safety and tolerability of rTMS

rTMS is generally very well tolerated, with most frequent side effects being mild headache, scalp discomfort or transient dizziness.^128^ Meta-analyses (mostly on depression cohort) report adverse event rates comparable to placebo and dropout rates similar to SSRIs, but without systemic side effects such as sexual dysfunction, gastrointestinal upset or orthostatic hypotension that are common and often disabling in Parkinson’s disease patients treated with antidepressants.^129,130^ Parkinson’s disease-specific safety data are also reassuring. A dedicated review^131^ identified 77 rTMS studies involving 1137 patients with Parkinson’s disease and found no reported seizures; adverse events were predominantly mild and transient, with a crude risk of 4.0% per patient and 0.39% per session. Reported events included headache, scalp pain, tinnitus, nausea and muscle jerks. Overall, these findings support rTMS as a relatively safe option in medically complex populations, although safety reporting in Parkinson’s disease studies remains less extensive and less standardized than in larger depression cohorts.

That said, safety reporting in device trials can be inconsistent, and underreporting of adverse events is possible. Real-world experience provides reassurance: in large rTMS clinics serious complications such as seizures or syncope occur in fewer than 0.1% of patients, aligning with international safety guidelines.^128^ This combination of trial data and clinical experience supports the view that rTMS carries a low complication burden, while highlighting the need for rigorous and standardized safety monitoring in future Parkinson’s disease-specific studies.

#### Summary, needs and future directions

rTMS offers a promising avenue for developing new therapeutic strategies in Parkinson’s disease. Beyond its established role in improving motor symptoms via HF stimulation over M1, it may help prevent motor complications—such as dyskinesias or fluctuations—by reducing medication needs. It also presents a non-pharmacological alternative for patients ineligible for DBS or advanced therapies due to medical or surgical contraindications. Given its favourable safety profile, pragmatic, well-powered trials are needed to determine rTMS’s optimal clinical use. Importantly, one of the central aims of this review was to highlight the marked heterogeneity of Parkinson’s disease rTMS literature: studies are mostly of small sample size, with variations in stimulation frequency, cortical target, laterality, coil configuration, treatment duration and maintenance design. These variables limit firm protocol-specific recommendations; however, the repeated emergence of positive signals across studies suggests that the field is detecting real therapeutic effects rather than isolated findings.

Advances in human neuroscience have clarified how specific neuropsychiatric symptoms stem from distinct circuit dysfunctions, supporting network-based neuromodulation. In practice, this framework could be implemented by combining symptom-specific phenotyping with structural and functional neuroimaging, as well as connectivity-informed targeting strategies, to identify the cortical entry points most closely linked to the relevant dysfunctional networks and thereby refine patient selection and stimulation protocols. This principle, established in psychiatric disorders like depression and OCD, is gaining traction in movement disorders including Parkinson’s disease and dystonia. Very recently, a circuit-based approach revealed that rTMS can be refined for the treatment of motor symptoms in Parkinson’s disease.^132^ Given Parkinson’s disease’s multisystem nature, a personalized, multitarget approach may be necessary to address its broad symptom range. Future trials should consider symptomatic heterogeneity, use clear inclusion criteria and apply multidimensional outcomes. For instance, HF-rTMS of the left DLPFC shows promise for cognition, depression, fatigue and apathy—but separate trials in defined populations are needed to isolate direct from indirect effects. As evidence grows for symptom-specific and shared network nodes, it is likely that different protocols will be required even within the same region, or that similar stimulation across distinct targets may produce benefits. Accelerated and connectivity-guided rTMS, emerging in affective disorders, could help rapidly identify and differentiate therapeutic responses in Parkinson’s disease.

Finally, current evidence does not exclude the possibility of disease-modifying effect stemming from symptom-oriented therapies such as those described here. Whether direct or indirect, this potential benefit should not be overlooked. Improved motor function is linked to higher levels of physical activity,^110-112^ and exercise-based interventions have shown possible neuroprotective effects.^133-135^ As standard Parkinson’s disease care must integrate symptomatic treatment for both motor and non-motor symptoms, incorporating physical, social and occupational activity within a multidisciplinary framework remains essential. Given that Parkinson’s disease treatment requires a combination of pharmacological and non-pharmacological approaches, it remains unclear whether their effects are merely additive or potentially multiplicative. Emerging research suggests that rTMS could serve as an adjuvant therapy with testable additive or multiplicative effects, as hinted by studies on pharmacologically enhanced rTMS^136^ and rTMS-augmented motor rehabilitation.^137^ Indeed, and despite the challenges of conducting complex non-pharmacological trials in Parkinson’s disease, the growing body of positive findings from small rTMS studies cannot be ignored. A coordinated academic research strategy that leverages technological advancements and clinical relevance is needed to generate high-quality evidence and clarify if rTMS is a meaningful therapeutic tool in Parkinson’s disease.

## Supplementary Material

fcag346_Supplementary_Data

## Data Availability

Data sharing is not applicable to this article as no new data were created or analysed in this study.
