# Supplementary material for: Repetitive transcranial magnetic stimulation as adjunctive treatment in Parkinson’s disease: are we missing opportunities? An umbrella and scoping review
Source: Brain Commun. 2026 Sep 9;8(5):fcag346. doi: 10.1093/braincomms/fcag346 (PMC13621913; doi:10.1093/braincomms/fcag346)
Supplement: Supplementary file 1 [file fcag346_supplementary_data.docx]

**Supplementary Table 1: Methodological quality assessment of included systematic reviews and meta-analyses using AMSTAR 2.** Each review was assessed across the 16 AMSTAR 2 domains, and an overall confidence rating was assigned according to AMSTAR 2 guidance. Abbreviations: **Y**, yes; **PY**, partial yes; **N**, no; **NA**, not applicable (no meta-analysis conducted).

| **Study** | **1** | **2** | **3** | **4** | **5** | **6** | **7** | **8** | **9** | **10** | **11** | **12** | **13** | **14** | **15** | **16** | **Overall** |
| --- | --- | --- | --- | --- | --- | --- | --- | --- | --- | --- | --- | --- | --- | --- | --- | --- | --- |
| Wang et al., 2024^1^ | Y | Y | Y | PY | Y | Y | Y | PY | Y | N | Y | N | Y | Y | Y | Y | Moderate |
| Qiu et al., 2023^2^ | Y | Y | Y | PY | Y | Y | N | PY | Y | N | Y | Y | Y | Y | Y | Y | Low |
| Zhang et al., 2022^3^ | Y | Y | Y | PY | N | Y | N | Y | Y | N | Y | Y | Y | Y | Y | Y | Low |
| Yang et al., 2018^4^ | Y | N | Y | PY | N | Y | N | PY | N | N | Y | N | N | Y | Y | Y | Critically low |
| Madrid & Benninger, 2021^5^ | PY | N | Y | PY | Y | N | N | PY | PY | N | NA | NA | Y | Y | NA | Y | Critically low |
| Cheng et al., 2021^6^ | Y | N | Y | PY | Y | Y | N | PY | PY | N | Y | N | N | Y | Y | Y | Critically low |
| Xia et al., 2023^7^ | Y | Y | Y | PY | Y | Y | N | PY | PY | N | NA | NA | Y | Y | NA | Y | Low |
| Dong et al., 2023^8^ | Y | Y | Y | PY | Y | N | N | PY | Y | N | Y | N | Y | Y | Y | Y | Low |
| Zhang et al., 2024^9^ | Y | Y | Y | PY | Y | Y | N | PY | Y | N | Y | N | Y | Y | Y | Y | Low |
| Nardone et al., 2020^10^ | PY | N | N | N | N | N | N | PY | N | N | NA | NA | N | Y | NA | Y | Critically low |
| Kim et al., 2019^11^ | Y | N | Y | PY | Y | N | N | PY | PY | N | Y | N | N | Y | N | Y | Critically low |
| Xie et al., 2020^12^ | Y | N | Y | PY | Y | Y | N | PY | PY | N | Y | Y | Y | Y | Y | Y | Critically low |
| Delgado-Alvarado et al., 2020^13^ | PY | N | Y | PY | N | Y | N | PY | PY | N | NA | NA | Y | Y | NA | Y | Critically low |
| Deng et al., 2022^14^ | Y | Y | Y | PY | Y | Y | N | PY | Y | N | Y | N | Y | Y | Y | Y | Low |
| Liu et al., 2024^15^ | Y | Y | Y | PY | Y | Y | N | PY | Y | N | Y | N | Y | Y | Y | Y | Low |
| Yang et al., 2024^16^ | Y | Y | Y | PY | Y | Y | N | PY | Y | N | Y | N | Y | Y | Y | Y | Low |

**Supplementary Table 2: Characteristics and main findings of rTMS studies targeting levodopa-induced dyskinesia in Parkinson’s disease**

Abbreviations: AIMS, Abnormal Involuntary Movement Scale; CAPSIT-PD, Core Assessment Program for Surgical Interventional Therapies in Parkinson’s Disease; CDRS, Clinical Dyskinesia Rating Scale; cTBS, continuous theta-burst stimulation; DLPFC, dorsolateral prefrontal cortex; IFC, inferior frontal cortex; iTBS, intermittent theta-burst stimulation; M1, primary motor cortex; preSMA, pre-supplementary motor area; RCT, randomized controlled trial; SMA, supplementary motor area; UDysRS, Unified Dyskinesia Rating Scale; UPDRS-IV, Unified Parkinson’s Disease Rating Scale Part IV.

| **Study** | **Design** | **n** | **State** | **Target** | **Protocol** | **Sessions** | **Main dyskinesia outcome** | **Main finding** |
| --- | --- | --- | --- | --- | --- | --- | --- | --- |
| Koch et al., 2005^17^ | Crossover | 8 | Acute | Bilateral SMA | 1 Hz, 900 pulses | 1 | AIMS | Transient improvement at 0–15 min; absent at 30 min |
| Brusa et al., 2006^18^ | Crossover | 10 | Acute | Bilateral SMA | 1 Hz, 900 pulses | 1 | AIMS | Transient improvement at 0–15 min; absent at 30 min |
| Wagle-Shukla et al., 2007^19^ | Single-arm | 6 | Chronic | Bilateral SMA | 1 Hz, 900 pulses | 10 | CAPSIT-PD dyskinesia | Reduction in mean/maximal dyskinesia |
| Rektorova et al., 2008^20^ | Crossover | 6 | Chronic | DLPFC / motor cortex | 10 Hz, 1350 pulses | 5 | UPDRS-IV | Trend for DLPFC benefit; no motor cortex effect |
| Koch et al., 2009^21^ | Parallel | 20 | Chronic + acute | Cerebellum | cTBS, 600 pulses/side | 10 | CAPSIT-PD dyskinesia | Reduction in dyskinesia |
| Filipović et al., 2009^22^ | Crossover | 10 | Chronic | Motor cortex | 1 Hz, 1800 pulses | 5 | CDRS | Reduction, particularly dystonic dyskinesia |
| Brusa et al., 2012^23^ | Crossover | 8 | Chronic | Cerebellum | Bilateral cTBS, 600 pulses/side | 5 | CAPSIT-PD dyskinesia | Reduction in dyskinesia |
| Sayın et al., 2014^24^ | RCT | 17 | Chronic | Bilateral SMA | 1 Hz, 1800 pulses | 10 | AIMS | Reduction in dyskinesia |
| Cerasa et al., 2015^25^ | Crossover | 11 | Acute | Right IFC | cTBS, 600 pulses | 1 | AIMS | Reduction after right IFC cTBS; no effect with iTBS or M1 cTBS |
| Flamez et al., 2016^26^ | Crossover | 9 / 6 | Acute + chronic | Bilateral M1 | 1 Hz, 1000 pulses | 1 or 5 | AIMS | No effect |
| Ponzo et al., 2016^27^ | Crossover | 10 | Acute | IFC | cTBS, 600 pulses | 1 | AIMS | Reduction in dyskinesia |
| Sanna et al., 2020^28^ | Single-arm | 11 | Acute | Cerebellum | cTBS, 600 pulses | 1 | CAPSIT-PD dyskinesia | Reduction in dyskinesia |
| Lohse et al., 2020^29^ | Randomized crossover | 17 | Acute | preSMA | 1 Hz, 1800 pulses | 1 | UDysRS | Reduction in dyskinesia |
| Flamez et al., 2021^30^ | Crossover | 9 | Acute | Right preSMA | 1 Hz, 1000 pulses | 1 | AIMS | No clinical effect |

**Supplementary Figure 1. Risk-of-bias assessment of studies evaluating repetitive transcranial magnetic stimulation for levodopa-induced dyskinesia in Parkinson’s disease**. Top: traffic-light plot showing study-level judgments across RoB2 domains. Bottom: summary plot showing the proportion of studies rated as low risk, some concerns, or high risk in each domain.

**Supplementary Table 3: Characteristics and main findings of rTMS studies targeting dysarthria in Parkinson’s disease**

Abbreviations: 3F Test, test of orofacial function/facial expression, speech, and tongue movements; DLPFC, dorsolateral prefrontal cortex; LSVT, Lee Silverman Voice Treatment; M1, primary motor cortex; n, sample size/number of participants; OFM1, orofacial primary motor cortex; STG, superior temporal gyrus; V-RQOL, Voice-Related Quality of Life

| **Study** | **Design** | **n** | **Target / protocol** | **Sessions** | **Main outcome** | **Main finding** |
| --- | --- | --- | --- | --- | --- | --- |
| **Dias et al., 2006**^31^ | Parallel RCT + single-arm cohort | 22 / 8 | Left DLPFC, 15 Hz vs sham; M1-mouth, 5 Hz open-label | 10 | Speech analysis; V-RQOL | DLPFC: V-RQOL/mood improvement but no objective speech effect; M1-mouth: improved fundamental frequency and voice intensity |
| **Brabenec et al., 2021**^32^ | Parallel sham-controlled RCT | 33 | Right STG, 1 Hz vs sham | 10 | 3F Test, phonetics score | Significant time-by-group interaction favouring real rTMS |
| **Li et al., 2023**^33^ | RCT / combined therapy study | 20 | LSVT-LOUD alone vs LSVT + TMS over left/right laryngeal M1 | 16 | Acoustic intonation analysis | Intonation changes were mainly attributed to LSVT, without clear added TMS effect |
| **Brabenec et al., 2019**^34^ | Randomized crossover | 16 | Right STG 1/10 Hz; OFM1 10 Hz; vertex control | 1 | Acoustic analysis | 1 Hz right STG improved an articulation-related acoustic parameter |
| **Eliasova et al., 2013**^35^ | Crossover | 12 | Left orofacial M1, 10 Hz; left DLPFC, 10 Hz | 1 | Acoustic analysis; 3F Test | M1 stimulation improved selected voice/speech measures; DLPFC showed no clear effect |

**Supplementary Figure 2. Risk-of-bias assessment of studies evaluating repetitive transcranial magnetic stimulation for dysarthria in Parkinson’s disease**. Top: traffic-light plot showing study-level judgments across RoB2 domains. Bottom: summary plot showing the proportion of studies rated as low risk, some concerns, or high risk in each domain.

**Supplementary Table 4: Characteristics and main findings of rTMS studies targeting dysphagia in Parkinson’s disease**

Abbreviations: A-DHI, Arabic-Dysphagia Handicap Index; fMRI, functional magnetic resonance imaging; M1, primary motor cortex; RMT, resting motor threshold;; UPDRS-III, Unified Parkinson’s Disease Rating Scale Part III.

| **Study** | **Design** | **n** | **Target / protocol** | **Sessions** | **Main outcome** | **Main finding** |
| --- | --- | --- | --- | --- | --- | --- |
| **Khedr et al., 2017**^36^ | Double-blind parallel-group RCT | 33 | Bilateral sequential M1 hand area; 20 Hz; 90% RMT; 2000 pulses/hemisphere | 10 + monthly boosters | A-DHI; video-fluoroscopy; UPDRS-III | Improved A-DHI and video-fluoroscopy measures versus sham; effect maintained to 3 months with booster sessions |
| **Huang et al., 2022**^37^ | Case-control / uncontrolled treatment study | 38 | Unilateral M1; 10 Hz; 90% RMT; 1200 pulses | 10 | Dysphagia Handicap Index; fMRI swallowing task | Improved subjective swallowing measures after rTMS, with changes in caudate/parahippocampal activation |

**Supplementary Figure 3. Risk-of-bias assessment of studies evaluating repetitive transcranial magnetic stimulation for dysphagia in Parkinson’s disease**. Traffic-light plot showing study-level judgments across RoB2 domains.

**Supplementary Table 5: Characteristics and main findings of rTMS studies targeting sleep in Parkinson’s disease**

Abbreviations: DLPFC, dorsolateral prefrontal cortex; PDSS, Parkinson’s Disease Sleep Scale; PSG, polysomnography; PSQI, Pittsburgh Sleep Quality Index; REM, rapid eye movement; RMT, resting motor threshold.

| **Study** | **Design** | **n** | **Target / protocol** | **Sessions** | **Main outcome** | **Main finding** |
| --- | --- | --- | --- | --- | --- | --- |
| **van Dijk et al., 2009**^38^ | Randomized active-stimulation study | 13 | Parietal cortex, 5 Hz, 80% RMT, 500 pulses | 10 | Actigraphy | Improved sleep efficiency and fragmentation |
| **Arias et al., 2010**^39^ | Double-blind randomized placebo-controlled trial | 18 | Vertex, 1 Hz, 90% RMT, 100 pulses | 10 | PDSS; actigraphy | No significant sleep benefit |
| **Zhuang et al., 2020**^40^ | Randomized single-blind sham-controlled parallel trial | 33 | Right DLPFC, 1 Hz, 110% RMT, 1200 pulses | 10 | PSQI | Improved sleep quality at 1 and 3 months |
| **Zhang et al., 2022**^41^ | Randomized sham-controlled trial | 25 | Right DLPFC, 1 Hz | 10 | Epworth Sleepiness Scale | Improved excessive daytime sleepiness |
| **Khedr et al., 2024**^42^ | Randomized clinical trial, pilot | 24 | Bilateral parietal cortex, 20 Hz, 80% RMT, 2000 × 2 pulses | 10 | PDSS; PSG | Improved subjective and objective sleep measures |
| **Wu et al., 2024**^43^ | Randomized double-blind sham-controlled trial | 67 | Right DLPFC, 1 Hz | 10 | PSG; sleep scales | Improved objective sleep architecture, including REM proportion |

**Supplementary Figure 4. Risk-of-bias assessment of studies evaluating repetitive transcranial magnetic stimulation for sleep in Parkinson’s disease**. Top: traffic-light plot showing study-level judgments across RoB2 domains. Bottom: summary plot showing the proportion of studies rated as low risk, some concerns, or high risk in each domain.

**References**

**1.** Wang Y, Ding Y, Guo C. Assessment of noninvasive brain stimulation interventions in Parkinson's disease: a systematic review and network meta-analysis. *Sci Rep.* 2024;14(1):14219. doi:10.1038/s41598-024-64196-0

**2.** Qiu Y, Yin Z, Wang M, et al. Motor function improvement and acceptability of non-invasive brain stimulation in patients with Parkinson's disease: a Bayesian network analysis. *Front Neurosci.* 2023;17:1212640. doi:10.3389/fnins.2023.1212640

**3.** Zhang W, Deng B, Xie F, et al. Efficacy of repetitive transcranial magnetic stimulation in Parkinson's disease: A systematic review and meta-analysis of randomised controlled trials. *EClinicalMedicine.* 2022;52:101589. doi:10.1016/j.eclinm.2022.101589

**4.** Yang C, Guo Z, Peng H, et al. Repetitive transcranial magnetic stimulation therapy for motor recovery in Parkinson's disease: A Meta-analysis. *Brain Behav.* 2018;8(11):e01132. doi:10.1002/brb3.1132

**5.** Madrid J, Benninger DH. Non-invasive brain stimulation for Parkinson's disease: Clinical evidence, latest concepts and future goals: A systematic review. *J Neurosci Methods.* 2021;347:108957. doi:10.1016/j.jneumeth.2020.108957

**6.** Cheng B, Zhu T, Zhao W, et al. Effect of Theta Burst Stimulation-Patterned rTMS on Motor and Nonmotor Dysfunction of Parkinson's Disease: A Systematic Review and Metaanalysis. *Front Neurol.* 2021;12:762100. doi:10.3389/fneur.2021.762100

**7.** Xia Y, Wang M, Zhu Y. The Effect of Cerebellar rTMS on Modulating Motor Dysfunction in Neurological Disorders: a Systematic Review. *Cerebellum.* 2023;22(5):954-972. doi:10.1007/s12311-022-01465-6

**8.** Dong K, Zhu X, Xiao W, et al. Comparative efficacy of transcranial magnetic stimulation on different targets in Parkinson's disease: A Bayesian network meta-analysis. *Front Aging Neurosci.* 2022;14:1073310. doi:10.3389/fnagi.2022.1073310

**9.** Zhang Y, Liu S, Xu K, et al. Non-pharmacological therapies for treating non-motor symptoms in patients with Parkinson's disease: a systematic review and meta-analysis. *Front Aging Neurosci.* 2024;16:1363115. doi:10.3389/fnagi.2024.1363115

**10.** Nardone R, Versace V, Brigo F, et al. Transcranial magnetic stimulation and gait disturbances in Parkinson's disease: A systematic review. *Neurophysiol Clin.* 2020;50(3):213-225. doi:10.1016/j.neucli.2020.05.002

**11.** Kim YW, Shin IS, Moon HI, Lee SC, Yoon SY. Effects of non-invasive brain stimulation on freezing of gait in parkinsonism: A systematic review with meta-analysis. *Parkinsonism Relat Disord.* 2019;64:82-89. doi:10.1016/j.parkreldis.2019.02.029

**12.** Xie YJ, Gao Q, He CQ, Bian R. Effect of Repetitive Transcranial Magnetic Stimulation on Gait and Freezing of Gait in Parkinson Disease: A Systematic Review and Meta-analysis. *Arch Phys Med Rehabil.* 2020;101(1):130-140. doi:10.1016/j.apmr.2019.07.013

**13.** Delgado-Alvarado M, Marano M, Santurtún A, Urtiaga-Gallano A, Tordesillas-Gutierrez D, Infante J. Nonpharmacological, nonsurgical treatments for freezing of gait in Parkinson's disease: A systematic review. *Mov Disord.* 2020;35(2):204-214. doi:10.1002/mds.27913

**14.** Deng S, Dong Z, Pan L, et al. Effects of repetitive transcranial magnetic stimulation on gait disorders and cognitive dysfunction in Parkinson's disease: A systematic review with meta-analysis. *Brain Behav.* 2022;12(8):e2697. doi:10.1002/brb3.2697

**15.** Liu Z, Wen X, Xie X, et al. The effects of transcranial magnetic stimulation for freezing of gait in Parkinson's disease: a systematic review and meta-analysis of randomized controlled trials. *Front Aging Neurosci.* 2024;16:1304852. doi:10.3389/fnagi.2024.1304852

**16.** Yang Y, Yan Z, Chang W, Ding J, Xu H. Effect of different modalities of transcranial magnetic stimulation on Parkinson's patients cognitive impairment and long-term effectiveness: a systematic review and network meta-analysis. *Front Neurosci.* 2024;18:1354864. doi:10.3389/fnins.2024.1354864

**17.** Koch G, Brusa L, Caltagirone C, et al. rTMS of supplementary motor area modulates therapy-induced dyskinesias in Parkinson disease. *Neurology.* 2005;65(4):623-625. doi:10.1212/01.wnl.0000172861.36430.95

**18.** Brusa L, Versace V, Koch G, et al. Low frequency rTMS of the SMA transiently ameliorates peak-dose LID in Parkinson's disease. *Clinical Neurophysiology.* 2006;117(9):1917-1921. doi:10.1016/j.clinph.2006.03.033

**19.** Wagle-Shukla A, Angel MJ, Zadikoff C, et al. Low-frequency repetitive transcranial magnetic stimulation for treatment of levodopa-induced dyskinesias. *Neurology.* 2007;68(9):704-705. doi:10.1212/01.wnl.0000256036.20927.a5

**20.** Rektorova I, Sedlackova S, Telecka S, Hlubocky A, Rektor I. Dorsolateral Prefrontal Cortex: A Possible Target for Modulating Dyskinesias in Parkinson's Disease by Repetitive Transcranial Magnetic Stimulation. *Int J Biomed Imaging.* 2008;2008(1). doi:10.1155/2008/372125

**21.** Koch G, Brusa L, Caltagirone C, et al. Cerebellar magnetic stimulation decreases levodopa-induced dyskinesias in Parkinson disease. *Neurology.* 2009;73(2):113-119. doi:10.1212/WNL.0b013e3181ad5387

**22.** Filipović SR, Rothwell JC, van de Warrenburg BP, Bhatia K. Repetitive transcranial magnetic stimulation for levodopa-induced dyskinesias in Parkinson's disease. *Movement Disorders.* 2009;24(2):246-253. doi:10.1002/mds.22348

**23.** Brusa L, Ceravolo R, Kiferle L, et al. Metabolic changes induced by theta burst stimulation of the cerebellum in dyskinetic Parkinson's disease patients. *Parkinsonism Relat Disord.* 2012;18(1):59-62. doi:10.1016/j.parkreldis.2011.08.019

**24.** Sayın S, Çakmur R, Yener GG, Yaka E, Uğurel B, Uzunel F. Low-frequency repetitive transcranial magnetic stimulation for dyskinesia and motor performance in Parkinson's disease. *Journal of Clinical Neuroscience.* 2014;21(8):1373-1376. doi:10.1016/j.jocn.2013.11.025

**25.** Cerasa A, Koch G, Donzuso G, et al. A network centred on the inferior frontal cortex is critically involved in levodopa-induced dyskinesias. *Brain.* 2015;138(2):414-427. doi:10.1093/brain/awu329

**26.** Flamez A, Cordenier A, De Raedt S, et al. Bilateral low frequency rTMS of the primary motor cortex may not be a suitable treatment for levodopa-induced dyskinesias in late stage Parkinson's disease. *Parkinsonism Relat Disord.* 2016;22:62-67. doi:10.1016/j.parkreldis.2015.11.009

**27.** Ponzo V, Picazio S, Benussi A, et al. Altered inhibitory interaction among inferior frontal and motor cortex in l-dopa-induced dyskinesias. *Movement Disorders.* 2016;31(5):755-759. doi:10.1002/mds.26520

**28.** Sanna A, Follesa P, Puligheddu M, et al. Cerebellar continuous theta burst stimulation reduces levodopa-induced dyskinesias and decreases serum BDNF levels. *Neurosci Lett.* 2020;716:134653. doi:10.1016/j.neulet.2019.134653

**29.** Lohse A, Meder D, Nielsen S, et al. Low-frequency transcranial stimulation of pre-supplementary motor area alleviates levodopa-induced dyskinesia in Parkinson's disease: a randomized cross-over trial. *Brain Commun.* 2020;2(2). doi:10.1093/braincomms/fcaa147

**30.** Flamez A, Wu GR, Wiels W, et al. Opposite effects of one session of 1 Hz rTMS on functional connectivity between pre-supplementary motor area and putamen depending on the dyskinesia state in Parkinson's disease. *Clinical Neurophysiology.* 2021;132(4):851-856. doi:10.1016/j.clinph.2020.12.024

**31.** Dias AE, Barbosa ER, Coracini K, Maia F, Marcolin MA, Fregni F. Effects of repetitive transcranial magnetic stimulation on voice and speech in Parkinson's disease. *Acta Neurol Scand.* 2006;113(2):92-99. doi:10.1111/j.1600-0404.2005.00558.x

**32.** Brabenec L, Klobusiakova P, Barton M, et al. Non-invasive stimulation of the auditory feedback area for improved articulation in Parkinson's disease. *Parkinsonism Relat Disord.* 2019;61:187-192. doi:10.1016/j.parkreldis.2018.10.011

**33.** Eliasova I, Mekyska J, Kostalova M, Marecek R, Smekal Z, Rektorova I. Acoustic evaluation of short-term effects of repetitive transcranial magnetic stimulation on motor aspects of speech in Parkinson's disease. *J Neural Transm (Vienna).* 2013;120(4):597-605. doi:10.1007/s00702-012-0953-1

**34.** Brabenec L, Klobusiakova P, Simko P, Kostalova M, Mekyska J, Rektorova I. Non-invasive brain stimulation for speech in Parkinson's disease: A randomized controlled trial. *Brain Stimul.* 2021;14(3):571-578. doi:10.1016/j.brs.2021.03.010

**35.** Li Q, Millard K, Tetnowski J, Narayana S, Cannito M. Acoustic Analysis of Intonation in Persons With Parkinson's Disease Receiving Transcranial Magnetic Stimulation and Intensive Voice Treatment. *J Voice.* 2023;37(2):203-214. doi:10.1016/j.jvoice.2020.12.019

**36.** Khedr EM, Mohamed KO, Soliman RK, Hassan AMM, Rothwell JC. The Effect of High-Frequency Repetitive Transcranial Magnetic Stimulation on Advancing Parkinson's Disease With Dysphagia: Double Blind Randomized Clinical Trial. *Neurorehabil Neural Repair.* 2019;33(6):442-452. doi:10.1177/1545968319847968

**37.** Huang PL, Wang SJ, Sun RF, et al. Increased activation of the caudate nucleus and parahippocampal gyrus in Parkinson's disease patients with dysphagia after repetitive transcranial magnetic stimulation: a case-control study. *Neural Regen Res.* 2022;17(5):1051. doi:10.4103/1673-5374.324863

**38.** van Dijk KD, Møst EIS, Van Someren EJW, Berendse HW, van der Werf YD. Beneficial effect of transcranial magnetic stimulation on sleep in Parkinson's disease. *Mov Disord.* 2009;24(6):878-884. doi:10.1002/mds.22462

**39.** Arias P, Vivas J, Grieve KL, Cudeiro J. Double-blind, randomized, placebo controlled trial on the effect of 10 days low-frequency rTMS over the vertex on sleep in Parkinson's disease. *Sleep Med.* 2010;11(8):759-765. doi:10.1016/j.sleep.2010.05.003

**40.** Zhuang S, Wang FY, Gu X, et al. Low-Frequency Repetitive Transcranial Magnetic Stimulation over Right Dorsolateral Prefrontal Cortex in Parkinson's Disease. *Parkinsons Dis.* 2020;2020:7295414. doi:10.1155/2020/7295414

**41.** Zhang X, Zhuang S, Wu J, et al. Effects of repetitive transcranial magnetic stimulation over right dorsolateral prefrontal cortex on excessive daytime sleepiness in patients with Parkinson's disease. *Sleep Med.* 2022;100:133-138. doi:10.1016/j.sleep.2022.08.003

**42.** Khedr EM, Ahmed GK, Korayem MA, Elamary SASH, El-Kholy MM, Haridy NA. Short-Term Therapeutic Effect of Repetitive Transcranial Magnetic Stimulations of Sleep Disorders in Parkinson's Disease: A Randomized Clinical Trial (Pilot Study). *Brain Sci.* 2024;14(6). doi:10.3390/brainsci14060556

**43.** Wu J, Zhuang S, Zhang X, et al. Objective sleep enhancement in Parkinson's disease: A sham-controlled trial of low-frequency repetitive transcranial magnetic stimulation over the right dorsolateral prefrontal cortex. *Parkinsonism Relat Disord.* 2024;126:107050. doi:10.1016/j.parkreldis.2024.107050
